# Iranian Nurses’ Views on Barriers and Facilitators in Patient Education: A Cross-Sectional Study

**DOI:** 10.5539/gjhs.v7n5p288

**Published:** 2015-03-16

**Authors:** Somayeh Ramezanli, Zohreh Badiyepeymaie Jahromi

**Affiliations:** 1 Department of Nursing, Jahrom University of Medical Sciences, Jahrom, Iran

**Keywords:** patient education, nurses, barriers, facilitators

## Abstract

**Background:**

As a major factor in patient-centered care, patient education has a great impact on the quality of care provided by nurses; however, clinical nurses’ performance with regard to patient education is not satisfactory. This study is an attempt to investigate barriers and facilitators in patient education from nurses’ point of view.

**Methods:**

122 nurses at Jahrom University of Medical Sciences participated in this descriptive-cross sectional study. Sampling was based on the census method. The questionnaire used to collect data included questions about nurses’ demography, barriers (10 questions), and facilitators (10 questions) in patient education. The questionnaire was designed to be completed independently. To analyze the data, the researchers used descriptive statistics, including frequency, mean and standard deviation.

**Results:**

The highest scores related to barriers to patient education were: nurses’ insufficient knowledge, patients’ physical and emotional unpreparedness, and lack of a proper environment for education. The most important facilitators, on the other hand, were: enhancement of instructing nurses’ knowledge and skills, motivating nurses, and a step-by-step approach to patient education.

**Conclusion:**

It is important that nurses be prepared and motivated to train their patients. By satisfactory patient education on the part of nurses, patients will be more willing to cooperate in the treatment process.

## 1. Introduction

A major part of a nurse’s role, patient education consists of education and re-education patients with a view to enabling them (Nolan, Nolan, & Booth, 2001). Patient education, as an effective form of nursing care, must be practiced throughout hospitalization (Aziznejad, Zabihi, Hosseini, & Bijani, 2010): patients—and their families—need to be informed about their diseases and encouraged to actively participate in their care in order to improve their life quality (Marcolongo et al., 2001). Through training, patients will have the knowledge and skills needed to recover more quickly, e.g. awareness of the administered medicines, diets, drug interactions, and the symptoms that patients need to be familiar with and report to care-providers (Aziznejad et al., 2010).

Providing patients with enough information is a major factor in patient-centered care and determines the quality of care (Stoop, van’t Riet, & Berg, 2004). Patient education will increase patients’ satisfaction and decrease their anxiety, shorten length of stay (Hoving, Visser, Mullen, & van den Borne, 2010), and help them adapt to their diseases (H. Dehghani & K. Dehghani, 1997). According to the charter of patients’ rights, patients are entitled to precise information about their diagnosis, treatment and prognosis (Lillis, LeMone, LeBon, & Lynn, 2010). Obviously, patients’ limited awareness of their conditions will decrease their interest in the treatment process; result in lower satisfaction with their conditions, and increase length of stay (Edwardson, 2007). Various studies have proved the positive impact of patient education, and nurses are legally and ethically obliged to train patients; however, nurses do not perform their training role satisfactorily and medical centers neglect the importance of training. Edwadson (2007) claims that clinical nurses do not provide proper training, train patients only informally and in response to certain situations, and do not consider patient education as a priority. Few patients in the study of Deccache and Aujoulat (2001) stated that they had been given enough information and counsel about their health conditions. Similarly, in Weetch’s study (2003), the patients were dissatisfied with their training. An identification of the barriers and facilitators in patient education can improve nurses’ performance in this field.

### 1.1 Research Aim

This study is an attempt to identify the facilitating and inhibiting factors in patient education in clinical environments from nurses’ point of view.

## 2. Method

### 2.1 Participants

In this descriptive-periodical study, the sample was composed of nurses practicing in the internal, surgical, and intensive care units of hospitals affiliated to Jahrom University of Medical Sciences. Sampling was based on the census method. Participants were selected from the aforementioned units because patients normally have longer stays and nurses have the opportunity to have more contact with them. Nurses with less than a year’s experience were excluded from the study due to their limited experience. Out of the 150 questionnaires distributed, 143 were completed and returned; 20 of the returned questionnaires were eliminated due to incompleteness; the 123 remaining questionnaires were analyzed (response rate was 81%).

### 2.2 Procedure

The questionnaires were distributed by the co-researcher in various working shifts from June to August, 2013. The questionnaires, handed out all at once, were completed by the nurses at their leisure and returned.

### 2.3 Questionnaire

The tool used in this study was Tahery et al.’s (2011) patient education questionnaire, which is divided into three parts: demographic questions, barriers, and facilitators in patient education. The first part dealt with the participant’s demographic characteristics, part two with the barriers (10 items), and part three with the facilitators (10 items). Based on Likert 5 point scale, each question was rated from “very important” to “not important at all,” with the first answer worth 5 points and the latter, 1. The reliability of the questionnaire was verified based on test-retest: barriers (r=0.75), facilitators (r=0.78).

### 2.4 Ethical Considerations

This study has been approved by the Research Committee and Committee of Ethics at Jahrom University of Medical Sciences. Introduction documents issued by the Research Department were presented to the heads and nursing managers of the hospitals where the study took place. In accordance with the ethical standards, the objectives of the study were explained to the units under study and the participants were reassured that their information would remain confidential. Names were not required and each nurse was asked to give consent before they participated.

### 2.5 Data Analysis

To analyze the data, the researchers used descriptive statistics, including frequency, mean and standard deviation. Analysis of this study used the Statistical Package for Social Science SPSS version 16.0.

## 3. Results

The average age of the participating nurses was 32.36±7.71, and their average experience was 8.51±7.19. The majority of the nurses were female (82.1%), married (75.8%), and worked rotating shifts (86.2%) (Table 1).

**Table 1:** Demographic characteristics of nurses

Variables		N (%)
**sex**	male	22(17.88)
	female	101(82.11)
**Marital status**	single	30(24.1)
	married	94(75.8)
**Shift work**	rotating shifts	110(88)
	Fixed shifts	15(12)

From the nurses’ point of view, the most important barriers to patient education were, in order of importance: nurses’ limited knowledge and awareness, patients’ lack of physical and emotional preparation and lack of a proper environment for patient education (Table 2).

**Table 2:** Mean, standard deviation and prioritize barriers of patient education from the perspective of nurses

barriers	Mean ± SD	Priority
nurses’ limited knowledge and awareness	4.60±0.91	1
patients’ lack of physical and emotional preparation	4.57±0.51	2
lack of a proper environment for patient education	4.51±0.91	3
Lack of trust between patient and provider education	4.45±0.64	4
Lack of time and time limits	4.41±0.69	5
Patient education is not a priority compared to other nursing duties	4.35±0.71	6
Lack of resources and suitable educational tools	4.30±0.72	7
Shortage of nurses	4.22±0.66	8
discontinuity of nurses collaboration in patient education	4.12±0.81	9
discontinuity of patient education in different shift work	4.05±0.84	10

From among the 10 suggested facilitators of patient education, the following were given the highest scores: increasing nurses’ awareness and skills, motivating nurses, and a step-by-step implementation of patient education (Table 3).

**Table 3:** Mean, standard deviation and prioritize facilitators of patient education from the perspective of nurses

Facilitators	Mean ± SD	Priority
increasing nurses’ awareness and skills	4.58±0.64	1
motivating nurses	4.52±0.71	2
step-by-step implementation of patient education	4.49±0.67	3
greater emphasis of teachers and administrators on patient education	4.42±0.67	4
raising participation of Patient in teaching and learning	4.36±0.96	5
More importance to the evaluation of patient education	4.28±0.67	6
Use Educational assistance devices	4.30±0.72	7
Considering information pamphlet guide to teach a specific topic	4.10±0.66	8
Planning based on suitable time and place of education	4.07±0.78	9
Considering one or two nurses dedicated to patient education.	4.02±0.95	10

## 4. Discussion

In this study, the nurses believed that one of the major barriers to patient education is nurses’ insufficient knowledge and skills. This is in agreement with the results of similar studies (Tahery et al., 2011; Vahedian Azimi, Alhani, & Hedayat, 2011; Celik, Abma, Widdershoven, van Wijmen, & Klinge, 2008; Bernard et al., 2006). Aghakhani, sharifnia, Ranjbar, Rahbar and Beheshti (2012) cite the following as major barriers to patient education: nurses’ insufficient knowledge regarding the importance of such education, nurses’ belief that such education does not affect the quality of treatment, nurses’ lack of interest in this kind of education. Few nurses are familiar with the principles and methods of patient education; for instance, most nurses fail to communicate with their patients, which can inhibit the provision of patient education (Aziznejad et al., 2012). Vahedian Azimi et al. (2011) claim that nurses’ insufficient academic knowledge, and consequent failure to provide proper patient education, is mainly due to the defective education provided by nursing colleges.

Patients’ lack of physical and emotional preparation is another major barrier to patient education (Tahery et al., 2011; H. Dehghani & K. Dehghani, 1997). In Haddad’s study, patients’ poor health conditions and failure to make use of the training given by nurses are cited as barriers to patient education (17). Physical illnesses and the resultant anxiety can decline patients’ readiness to learn or prevent them from realizing the importance of education in their illnesses (Aziznejad et al., 2010). Similarly, in many studies, patients’ emotional and physical conditions are referred to as major barriers to patient education (Beverly, 2012; Daly, 2009; Tromp, Dulmen, & Weert, 2004; Green, Gross, Kernan, Wong, & Holmboe, 2003), which is in agreement with the findings of the present study. Beagley (2012) cites literacy, culture, language and physiological factors as barriers to care-givers’ presenting patients with information, and concludes that nurses need to consider patients’ various learning styles in order to select the most effective ways to prepare and teach them. In the study of Aghakhani et al. (2012) patients’ lack of interest in and indifference to learning are the most important barriers to patient education.

The nurses in this study referred to the inappropriateness of the clinical environment as a significant barrier to patient education, which is in agreement with the results of similar studies (Aziznejad et al., 2010; Rostami, Montazam, Ghahramanian, & Mirghafourvand, 2010; Marcum, Ridenour, Shaff, Hammons, & Taylor, 2001). In an appropriate environment, nurses and doctors have a good professional relationship, nurses play an active role in matters related to the hospital, managers consider and react to the clinical issues identified by nurses, and medical organizations invest in nurses’ continuous education and betterment of medical care (Aiken et al., 2011). Accordingly, managers in medical organizations—nursing managers, in particular—need to take measures to efficiently use the existing facilities, and also improve the conditions and provide the required tools for patient education.

Increasing the knowledge and skills of nurses who train patients is a major facilitator in patient education. Lupon et al. (2008) claim that nurses with vaster academic knowledge are more successful in patient education. Accordingly, well-informed nursing teachers, better education, and presentation of practical approaches to patient education based on patients’ various needs and modern developments in medical care will increase nursing students’ knowledge and skills, which will result in their better performance. Moreover, authorities, by meeting nurses’ educational needs and providing continuing educational programs and virtual education courses on various diseases and patients’ needs, can improve patient education.

Another facilitator is motivating nurses: Moridi, Khalidi, and Barfi (2009) introduce raising nurses’ interest in patient education and encouraging patients to learn as important facilitators in patient education. In the study of Aghakhani et al. (2012) the nurses mentioned that their attention to patient education did not result in job promotion for them. Other studies show that authorities and managers in medical care organizations do not motivate or prepare nurses for patient education (Lok et al., 2007). Ashghali-Farahani, Mohammadi, Ahmadi, Maleki and Hajizadeh (2009) refer to authorities’ disregard for patient education programs and lack of a systematic evaluation of such education as the most important barriers to patient education. Accordingly, managers’ consideration and regular evaluation of nurses’ role in patient education can motivate nurses to perform more carefully in this area. Should patient education not be given the required financial and spiritual support, it will pale into insignificance.

According to the results, a step-by-step implementation of patient education is another facilitator in such education: nurses should evaluate their patients’ conditions, identify their needs, and prioritize, plan, execute, and assess the required education based on their educational and cultural backgrounds, lengths of stay, and the existing facilities (Kalantari et al., 2011).

Since the study was limited to the hospitals affiliated to Jahrom University of Medical Sciences and the sample size was relatively small, it is not possible to freely generalize the results to other nurses in other areas and hospitals. Thus, it is necessary that nurses practicing in other hospitals be studied. It is also suggested that qualitative studies be conducted to complete the quantitative studies on patient education. Nursing managers’ views on the facilitators and barriers in patient education should also be studied and compared to the nurses’ views. Moreover, there is need for extensive studies to identify more efficient methods to enhance the quality of patient education.

## 5. Conclusion

Patient education is an established strategy to minimize hygienic risks and hospitalization costs. Nurses need to be prepared for such education so that they can choose the best approaches and have their patients participate in the process. By starting such preparation programs, managers can improve patient education, raise both patients’ and nurses’ awareness regarding the importance of patient education, popularizes patient education, and introduces scientific and practical techniques to practice it.
